# Recurrent Spontaneous Abortion (RSA) and Maternal *KIR* Genes: A Comprehensive Meta-Analysis

**DOI:** 10.5935/1518-0557.20190067

**Published:** 2020

**Authors:** Soheila Akbari, Farhad Shahsavar, Reza Karami, Fatemeh Yari, Khatereh Anbari, Seyyed Amir Yasin Ahmadi

**Affiliations:** 1 Department of Obstetrics and Gynecology, Lorestan University of Medical Sciences, Khorramabad, Iran; 2 Department of Immunology, Lorestan University of Medical Sciences, Khorramabad, Iran; 3 Department of Reproductive Health, Lorestan University of Medical Sciences, Khorramabad, Iran; 4 Social Determinants of Health Research Center, Lorestan University of Medical Sciences, Khorramabad, Iran; 5 Student Research Committee, Iran University of Medical Sciences, Tehran, Iran

**Keywords:** recurrent spontaneous abortion, killer-cell immunoglobulin-like receptor, human leukocyte antigen, meta-analysis

## Abstract

Natural killer cells (NKs) are the most important cells in the fetomaternal immune tolerance induced through interaction of maternal killer-cell immunoglobulin-like receptors (KIR) and fetal human leucocyte antigens (HLA). Hence, we intend to perform a meta-analysis on the role of maternal *KIR* genes diversity in recurrent spontaneous abortion (RSA). The present paper is a meta-analysis of previous genetic association studies and our previous original study. The results showed that *KIR3DL1* was a significantly protecting factor for RSA (*p*=0.044; OR=0.833 [0.698-0.995]; fixed effect model). *KIR2DS2* (*p*=0.034; OR=1.195 [1.013-1.408]; fixed effect model) and *KIR2DS3* (*p*=0.013; OR=1.246 [1.047-1.483]; fixed effect model) were significantly risk factors for RSA. For *KIR2DS1* there was a high heterogeneity and publication bias. Briefly, the inhibitory gene *KIR3DL1* was a protecting factor, and the activating genes *KIR2DS2* and *KIR2DS3* were risk factors for RSA. However, the effect sizes were not suitable. We suggest further studies on different causes of pregnancy loss, to find the role of *KIR2DS1*.

## INTRODUCTION

### Rationale

Recurrent spontaneous abortion (RSA) and pregnancy loss have different pathogeneses, consisting of genetic and chromosomal abnormalities (Hume & Chasen, 2015), environmental toxicities and oxidative stress (Gupta *et al*., 2007), infectious agents (Ambühl *et al*., 2016), hormonal causes, etc. Among them, immunological causes and their involving molecules are still controversial and unknown topics. The immune system is a fascinating system, one that does not normally reject the semi-allograft fetus. The immune system has two roles in implantation and pregnancy; preventing the formation of abnormal embryos, and protecting the fetomaternal interaction by releasing angiogenic factors, cytokines and adhesive molecules. The fascinating point is how a system can have two mutually exclusive features; protection and rejection. Indeed, the immune system is the bodyguard of the body through self- and non-self recognition. However, pregnancy is a semi-allograft transplantation. So the question is what the immune system does in this situation; rejection or protection (Akbari *et al*., 2018; Würfel, 2016)?! 

Immune tolerance is the best answer for the above question (Akbari *et al*., 2018; Würfel, 2016). Natural killer cells (NKs), which name is self-explanatory, are one of the most important lymphocytes in immune tolerance. They identify self-cells through their killer-cell immunoglobulin-like receptors (KIRs) expressed on their surface. The KIRs interact with their ligands, the human leukocyte antigens (HLAs) - the identification cards of self-cells. These interactions usually result in immune tolerance under normal conditions. Both *KIR* and *HLA* genes in human genome have loci (not locus), inherited as haplotypes. In addition, each gene in their loci is polymorphic. Thus, interaction of different KIR molecules with different HLA molecules results in different outcomes consisting of inhibitory and activating responses. *KIR* gene cluster is located on chromosome 19. This cluster has two types of genes, including 8 inhibitory and 6 activating genes, and 2 pseudogenes. Some of these genes exist in all individuals, like the *KIR2DL4*. From the viewpoint of medical anthropology, different people from different ethnicities have different KIR-HLA interactions (Alecsandru *et al*., 2014; Ashouri *et al*., 2016; Middleton *et al*., 2008; Norman *et al*., 2016; Solgi *et al*., 2011). 

HLA has two classes, I and II, and the class I can be further divided into classical and non-classical HLA. KIR2DL4 is an inhibitory KIR binding to the trophoblast HLA-G, which is a non-classical HLA. The combination KIR2DL4+HLA-G triggers the immune tolerance. Both *KIR2DL4* and *HLA-G* are polymorphic genes. Therefore, anthropological variations can contribute to implantation success and pregnancy maintenance. For example, HLA-G*01:03:01 is a risk factor for implantation failure; because its connection with KIR2DL4 is not sufficient to trigger inhibitory signals (Nardi et al., 2012). 

NKs may have the CD16 marker, which is the weapon of antibody-depended cell-mediated cytotoxicity (ADCC). Usually CD56^dim^ NKs are CD16^+^. So CD16^+^CD56^dim^ NKs are known as cytotoxic NKs, whereas CD16^-^CD56^bright^ NKs are known as immune-regulatory NKs (Ghafourian *et al*., 2015). About 90% of uterine NKs (UNKs) are immune-regulatory. In conclusion, UNKs are not usually cytotoxic for the embryo (Ghafourian et al., 2015; Sacks, 2015). 

### Objectives

As we mentioned above, KIR and HLA have different genes and interactions. KIR has 8 inhibitory (*2DL1, 2DL2, 2DL3, 2DL4, 2DL5, 3DL1, 3DL2* and *3DL3*) and 6 activating genes (*2DS1, 2DS2, 2DS3, 2DS4, 2DS5* and *3DS1*). Since the involving NKs in implantation of embryo are maternal, we intend to perform a meta-analysis on the role of maternal *KIR* genes diversity in RSA. Previously, Pereza *et al*. (2017) carried out a meta-analysis on different genes, including the *KIR*. Nevertheless, their studies were few and therefore our study can serve as an update for that meta-analysis.

## MATERIALS AND METHODS

### Study selection

For the present meta-analysis, we searched in scientific databases such as Web of Science, PubMed, Scopus, Google Scholar, etc. Our keywords were searched only among the titles. After exclusion of duplicates, all the eligible studies were used for qualitative systematic review.

### Eligibility criteria

Among the studies imported for qualitative systematic review, only the studies with available and enough numerical data were imported for the quantitative meta-analysis. Our original paper on this topic was manually added (Table 1) (Akbari *et al*., 2018). Performing *KIR* typing was the most important criterion.

**Table 1:** Data summery of the found articles.

Study	Witt *et al*., 2004		Wang *et al.,* 2007		Hong *et al.*, 2008		Hiby *et al*., 2008		Vargas *et al*., 2009		Faridi *et al.*, 2009		Khosravifar *et al*., 2011		Ozturk *et al.*, 2012		Djulejic *et al*., 2015		Dambaeva *et al*., 2016		Our original study	
Gene	RSA N=52	Control N=55	RSA N=73	Control N=68	RSA N=16	Control N=41	RSA N=95	Control N=269	RSA N=68	Control N=68	RSA N=205	Control N=224	RSA N=100	Control N=100	RSA N=40	Control N=90	RSA N=25	Control N=122	RSA N=139	Control N=195	RSA N=100	Control N=100
**2DL1**	52	55	73	68	8	21	92	258	63	64	141	215	97	95	40	89	24	115	135	189	93	95
P value (ED) a	1 (FET) b		1 (FET)		0.841 (-)		0.769 (FET) (-)		0.999 (FET) (-)		0.0001 (-)		0.720 (+)		1 (FET)		1 (FET)		1 (FET)		0.764 (-)	
**2DL2**	29	23	22	23	16	22	45	137	43	37	110	111	52	58	26	41	17	72	69	96		
*p* value (ED)	0.211 (+)		0.777 (-)		0.002 (+)		0.361 (-)		0.383 (+)		0.446 (+)		0.475 (-)		0.632 (+)		0.537 (+)		1			
**2DL3**	47	47	72	67	6	18	88	245	58	58	169	187	87	85	37	74	24	110	124	172		
*p* value (ED)	0.631 (+)		1 (FET)		0.887 (-)		0.806 (+)		1 (FET)		0.887 (-)		0.841 (+)		0.207 (+)		0.469 (FET) (+)		0.920 (+)			
**2DL4**																						
**2DL5**	16	20	35	28	5	12	36	148	37	33	127	151			32	56	4	50	79	103	58	60
*p* value (ED)	0.680 (-)		0.521 (+)		1 (FET)		0.005 (-)		0.610 (+)		0.238 (+)				0.072 (+)		0.032 (-)		0.537 (+)		0.887 (-)	
**3DL1**	50	48	73	67			88	256	64	63	120	191			36	81	24	117	125	185	93	95
*p* value (ED)	0.162 (FET) (+)		1 (FET)				0.502 (-)		0.999 (FET) (+)		0.0001 (-)				1 (FET)		1 (FET)		0.131 (-)		0.764 (-)	
**3DL2**																						
**3DL3**																						
**2DS1**	21	25	44	28	1	4	24	121	32	26	92	88	35	48	21	31	8	58	63	73	49	40
*p* value (ED)	0.740 (-)		0.035 (+)		1 (FET)		0.001 (-)		0.386 (+)		0.283 (+)		0.084 (-)		0.005 (+)		0.228 (-)		0.182 (+)		0.254 (+)	
**2DS2**	27	26	22	18	1	3	46	140	45	39	104	72	50	58	26	41	14	69	69	97	59	54
*p* value (ED)	0.777 (+)		0.764 (+)		1 (FET)		0.624 (-)		0.377 (+)		0.001 (+)		0.319 (-)		0.063 (+)		0.806 (+)		1		0.565 (+)	
**2DS3**	16	15	25	20	2	3	22	70	22	24	94	66			17	29	11	40	44	55	38	34
*p* value (ED)	0.824 (+)		0.622 (+)		0.613 (+)		0.680 (-)		0.862 (-)		0.0007 (+)				0.350 (+)		0.399 (+)		0.577 (+)		0.654 (+)	
**2DS4**	18	21	72	65	8	24	90	255	62	64	109	163			36	82	25	117	130	185	95	95
*p* value (ED)	0.862 (-)		0.352 (FET) (+)		0.777 (-)		1 (FET)		0.740 (-)		0.0001 (-)				1 (FET)		0.588 (FET) (+)		0.777 (-)		1	
**2DS5**	10	18	38	26	4	8	23	102	30	19	122	122			22	35	6	37	53	70	35	34
*p* value (ED)	0.171 (-)		0.139 (+)		0.722 (FET) (+)		0.021 (-)		0.074 (+)		0.337 (+)				0.129 (+)		0.698 (-)		0.764 (+)		1	
**3DS1**	17	20	38	32			24	121	34	23	162	116			16	37	7	46	62	77	41	40
*p* value (ED)	0.590 (-)		0.761 (+)				0.001 (-)		0.082 (+)		0.0001 (+)				0.920 (-)		0.488 (-)		0.409 (+)		1	
**2DP1**																						
**3DP1**																						
Study design	Case-control		Case-control		Case-control		Case-control		Case-control		Case-control		Case-control		Case-control		Case-control		Cohort for KIR2DS1		Case-control	
Genotyping method	PCR-SSP		PCR-SSP		PCR-SSP		PCR-SSP		PCR-SSO		PCR-SSP		PCR-SSP		PCR-SSO		PCR-SSP		PCR-SSO		PCR-SSP	
RSA definition	3 spontaneous abortion		3 spontaneous abortion		3 spontaneous abortion		3 spontaneous abortion		3 spontaneous abortion		3 spontaneous abortion		3 spontaneous abortion		A history of miscarriage		Any fertility problem		2 spontaneous abortion		3 spontaneous abortion	
Control definition	2 history of normal delivery		2 history of normal delivery		2 history of normal delivery		Any primiparous woman		2 history of normal delivery		2 history of normal delivery		1 history of normal delivery		2 history of normal delivery		Not mentioned		Not mentioned		2 history of normal delivery	
Place	Brazil		China		China		London		Brazil		India		Iranian		Mediterranean		Albania		America		Iran	
Ethnicity	Caucasian		Chinese		Chinese		Caucasian		Caucasian		Indian		Caucasian		Caucasian		Caucasian		Caucasian		Caucasian	
Study number in dendrogram	1		2		3		4		5		6		7		8		9		10		11	

a) ED stands for effect direction; the positive ones show risk factors and the negative ones show protecting factors.

b) FET stands for Fisher's exact test.

### Statistical analysis

To perform the present meta-analysis, we used the comprehensive meta-analysis version 2 software (Biostat, US). The analyses were carried out through a *p* value and individual sample size using fixed-effect and random-effect models. Since the *p* values were calculated using Yate's correction (or Fisher's exact test if necessary), the odds ratios (OR) (effect sizes) achieved from these *p* values were underestimated. This statistical protocol has been previously published (Anbari & Ahmadi *et al*., 2017). 

### Heterogeneity and publication bias

We used the *I^2^* scale and *I^2^*<50 was considered as homogeneity. In the cases of heterogeneity, we used the random-effect model. In order to find publication bias, we used funnel plots. If a study were to be find outside the funnel, it meant that its effect size was outside the expected 95% confidence interval (CI). In other words, its difference with other studies is statistically significant at *p*=0.05. Hence, a publication bias does not have necessarily a negative connotation. In the present study, a funnel plot *p* value < 0.05 means that the mentioned individual study is outside the funnel of 95% CI. 

### Additional analyses

**Figure 1:**
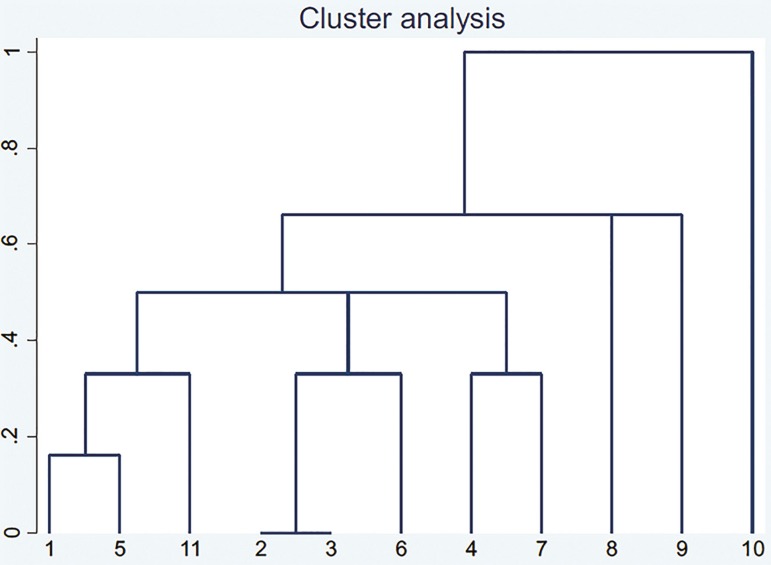
Cluster analysis of Table 2 based on complete linkage method. The numbers of studies are based on Tables 1 and 2.

In order to cluster the studies for meta-analysis, we designed a dendrogram using the STATA14 software (StataCorp LLC, US). This cluster analysis involved the complete linkage of binary variables (Table 2, Figure 1). 

**Table 2:** Dissimilarity matrix of studies' characteristics based on the below of Table 1

		Witt *et al*., 2004	Wang *et al*., 2007	Hong *et al*., 2008	Hiby *et al*., 2008	Vargas *et al*., 2009	Faridi *et al*., 2009	Khosravifar *et al*., 2011	Ozturk *et al*., 2012	Djulejic *et al*., 2015	Dambaeva *et al*. 2016	Our study
1	Witt *et al*., 2004	0	0.33	0.33	0.33	0.16	0.33	0.33	0.50	0.50	0.83	0.16
2	Wang *et al*., 2007	0.33	0	0	0.50	0.50	0.33	0.50	0.66	0.66	1	0.33
3	Hong *et al*., 2008	0.33	0	0	0.50	0.50	0.33	0.50	0.66	0.66	0.83	0.33
4	Hiby *et al*., 2008	0.33	0.50	0.50	0	0.50	0.50	0.33	0.66	0.50	0.83	0.33
5	Vargas *et al*., 2009	0.16	0.50	0.50	0.50	0	0.50	0.50	0.33	0.66	0.66	0.33
6	Faridi *et al*., 2009	0.33	0.33	0.33	0.50	0.50	0	0.50	0.66	0.66	1	0.33
7	Khosravifar *et al*., 2011	0.33	0.50	0.50	0.33	0.50	0.50	0	0.66	0.50	0.83	0.33
8	Ozturk *et al*., 2012	0.50	0.66	0.66	0.66	0.33	0.66	0.66	0	0.66	0.66	0.50
9	Djulejic *et al*., 2015	0.50	0.66	0.66	0.50	0.66	0.66	0.50	0.66	0	0.66	0.50
10	Dambaeva *et al*., 2016	0.83	1	0.83	0.83	0.66	1	0.83	0.66	0.66	0	0.83
11	Our original study	0.16	0.33	0.33	0.33	0.33	0.33	0.33	0.50	0.50	0.83	0

## RESULTS

### Eligible studies

Table 1 depicts the findings from the selected studies, in addition to our original case-control study, this table includes 11 studies. The *p* values were analyzed through Yate's correction (or fisher's exact test when necessary). Positive effect directions show each gene as a risk factor and negative effect directions show each gene as a protecting factor. Our cluster analysis showed that the study by Dambaeva *et al*. (2016) had a different design in comparison to other studies (Figure 1). Hence, it was excluded from the meta-analysis. At the end, 10 studies remained. 

### Meta-analysis

**Figure 2:**
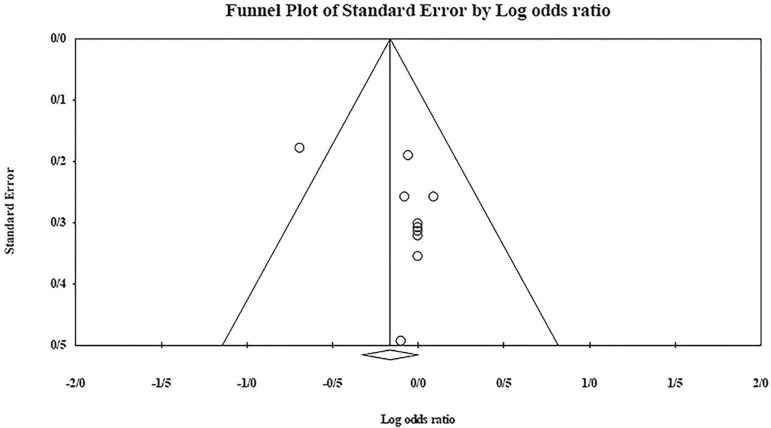
KIR2DL1 Funnel plot showing a significant bias for Faridi *et al*. (2009).

**Figure 3:**
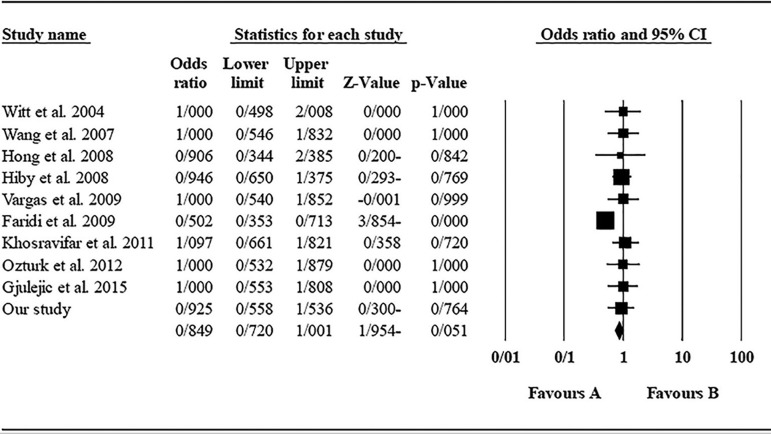
Forest plot of KIR2DL1 (fixed). Favours A shows protecting effect and favours B shows harmful effect (in all figures).

The role of *KIR2DL1* in RSA was not statistically significant (*p*=0.051; OR=0.849; fixed). Faridi *et al*. (2009) showed a significantly more protective effect of this gene in comparison to other studies (funnel plot *p* value <0.05) (Figures 2 and 3). 

**Figure 4:**
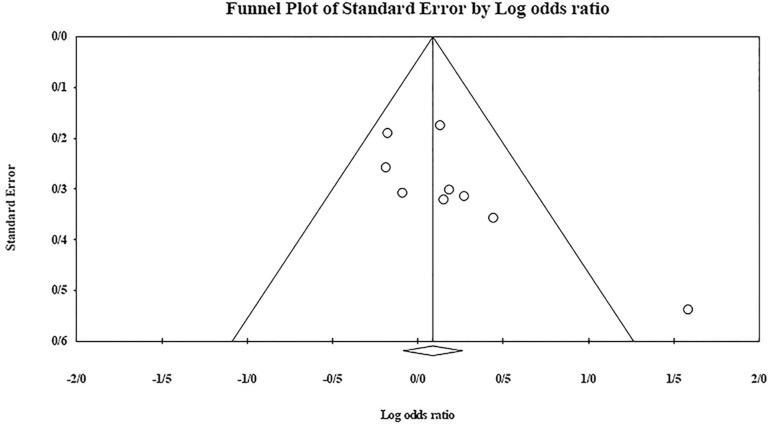
KIR2DL2 Funnel plot showing a significant bias for Hong *et al*. (2008).

**Figure 5:**
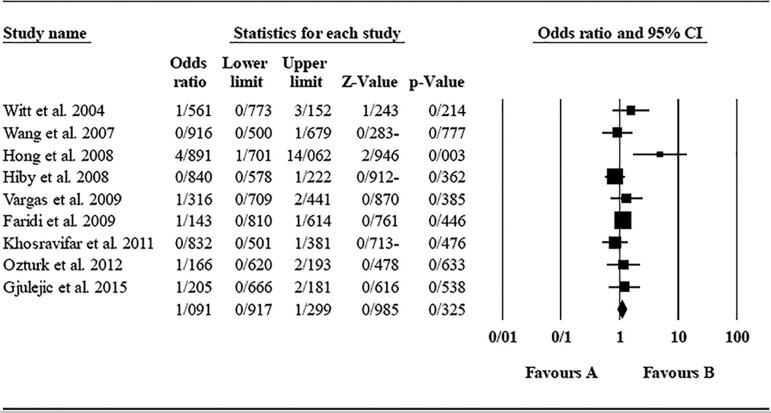
Forest plot of KIR2DL2 (fixed).

**Figure 6:**
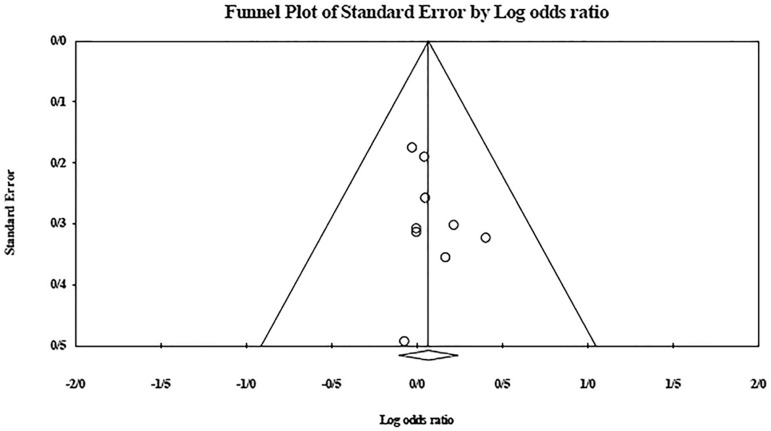
Funnel plot of KIR2DL3 shows no publication bias.

**Figure 7:**
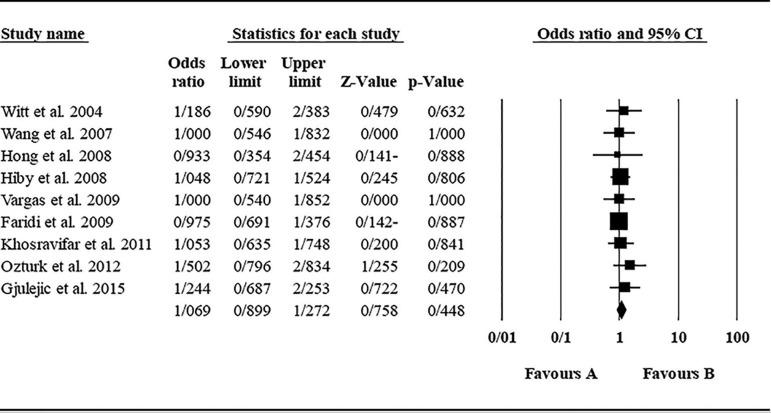
KIR2DL3 Forest plot (fixed).

**Figure 8:**
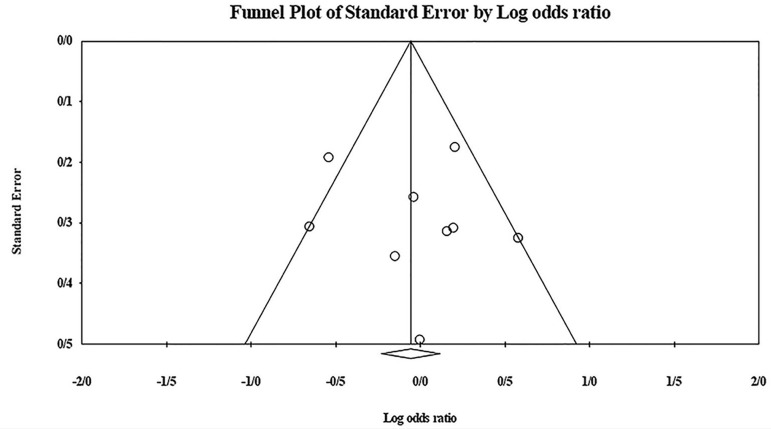
KIR2DL5 Forest plot showing a significant bias for Hiby et al. (2008) study.

**Figure 9:**
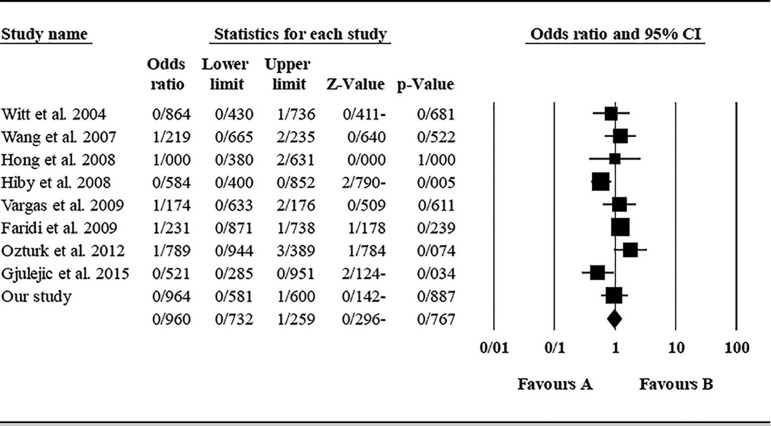
KIR2DL5 Forest plot (random).

The role of *KIR2DL2* in RSA was not statistically significant (*p*=0.325; OR=1.091; fixed). Hong *et al*. (2008) showed a significantly higher risk of this gene's effect in comparison to other studies (funnel plot *p* value <0.05) (Figures 4 and 5). The role of *KIR2DL3* in RSA was not statistically significant (*p*=0.448; OR=1.062; fixed). No publication bias was found based on the funnel plot (Figures 6 and 7). The role of *KIR2DL5* in RSA was not statistically significant (*p*=0.767; OR=0.960; random). Hiby *et al*. (2008) showed a significantly more protective effect of this gene in comparison to other studies (funnel plot *p* value <0.05) (Figures 8 and 9). 

**Figure 10:**
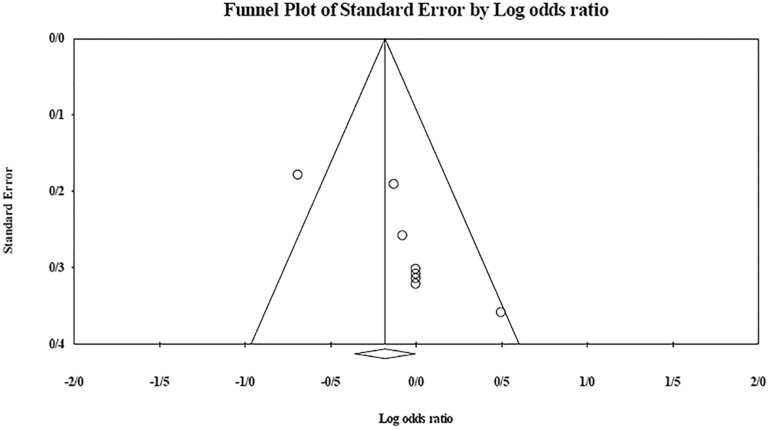
KIR3DL1 Funnel plot showing a significant bias for Faridi et al. (2009).

**Figure 11:**
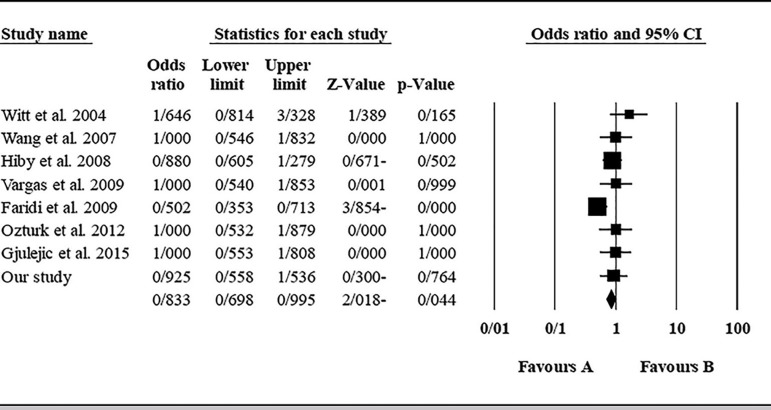
KIR3DL1 Forest plot (fixed).

**Figure 12:**
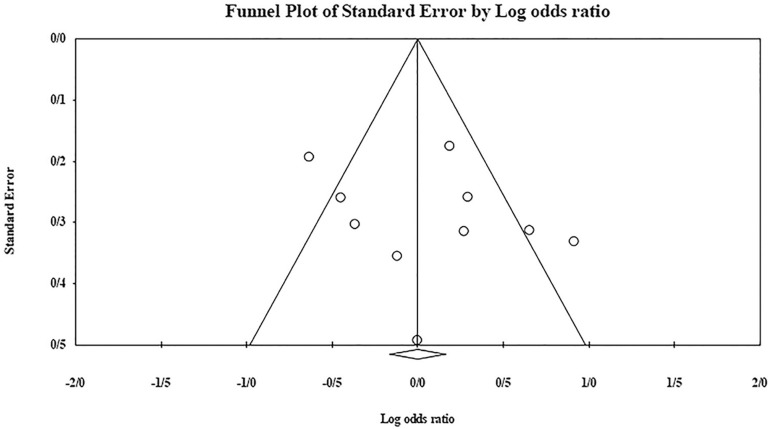
KIR2DS1 Funnel plot showing a huge publication bias which is inconclusive.

**Figure 13:**
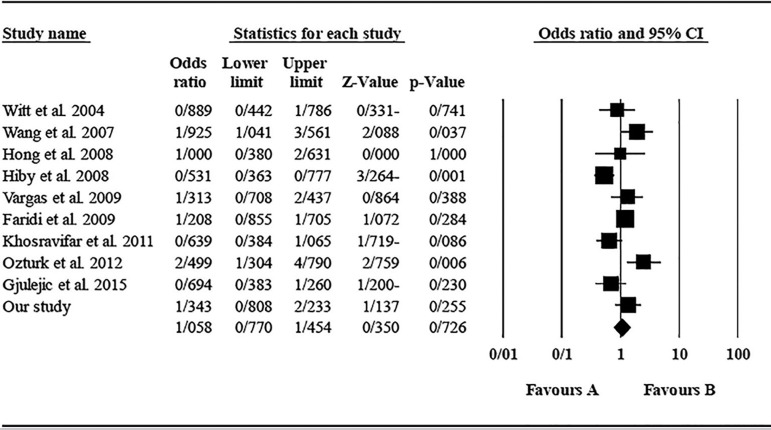
KIR2DS1 Forest plot (random).

**Figure 14:**
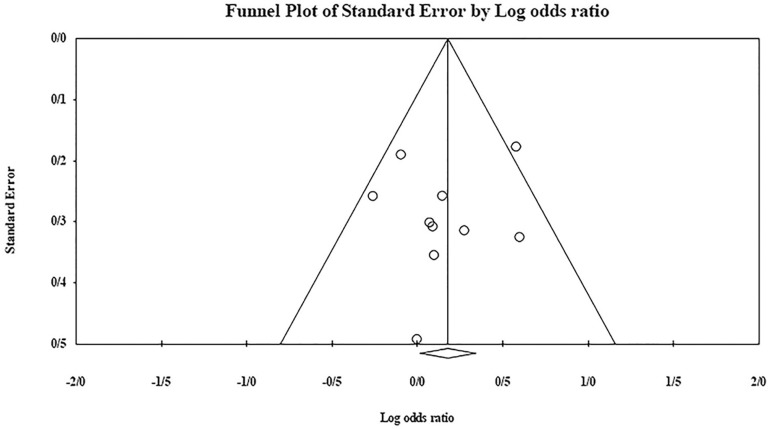
KIR2DS2 Funnel plot showing a significant bias for Faridi et al. (2009).

**Figure 15:**
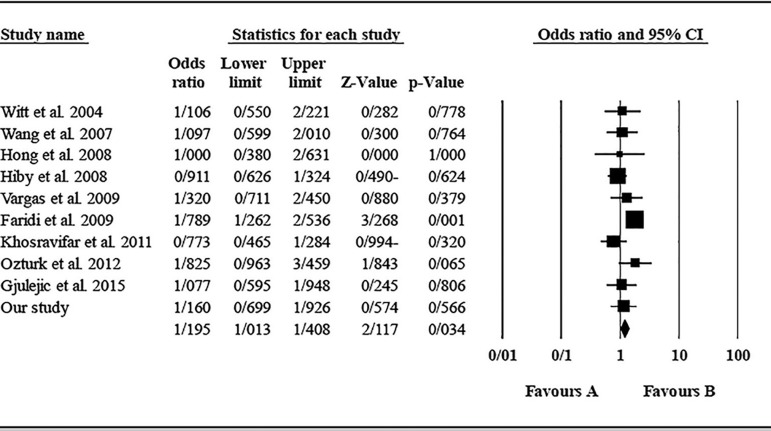
KIR2DS2 Forest plot (fixed).

**Figure 16:**
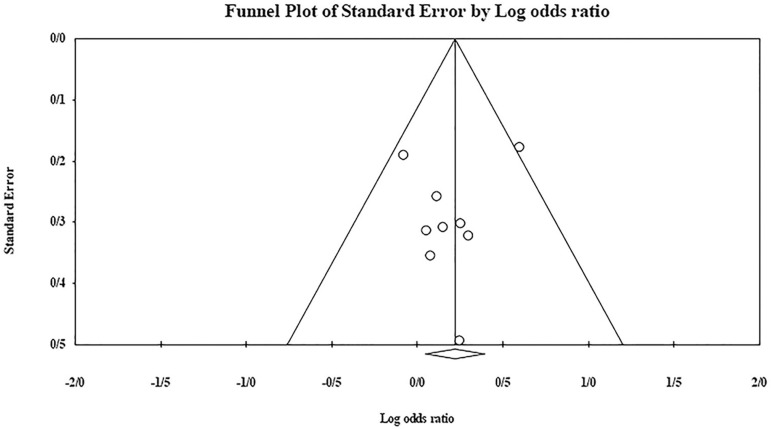
KIR2DS3 Funnel plot showing a rather significant bias for Faridi et al. (2009).

**Figure 17:**
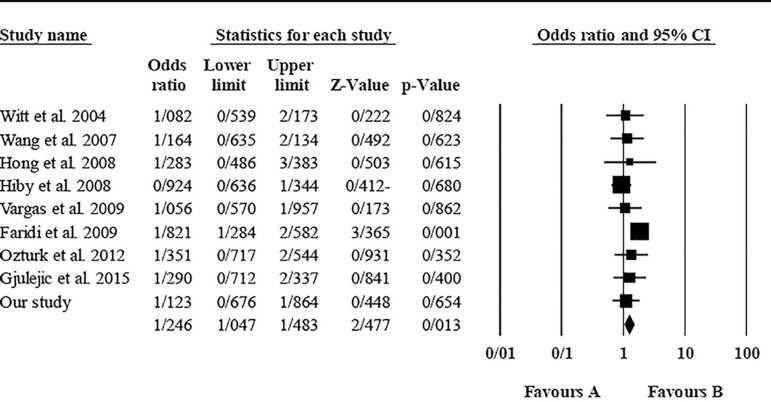
KIR2DS3 Forest plot (fixed).

The role of *KIR3DL1* in RSA was statistically significant (*p*=0.044*; OR=0.833; fixed). Faridi *et al*. (2009) showed a significantly more protective effect of this gene in comparison to other studies (*p*<0.05; based on funnel plot) (Figures 10 and 11). The role of *KIR2DS1* in RSA was not statistically significant (*p*=0.726; OR=1.056; random). Inconclusive publication bias was found for this analysis based on the funnel plot (Figures 12 and 13). The role of *KIR2DS2* in RSA was statistically significant (*p*=0.034*; OR=1.195; fixed). Faridi *et al.* (2009) study showed significantly more risk effect of this gene in comparison to other studies (funnel plot ** value <0.05) (Figures 14 and 15). The role of *KIR2DS3* in RSA was statistically significant (*p*=0.013*; OR=1.246; fixed). Faridi *et al.* (2009) showed significantly more risk effect of this gene in comparison to other studies (funnel plot *p* value <0.05) (Figures 16 and 17). 

**Figure 18:**
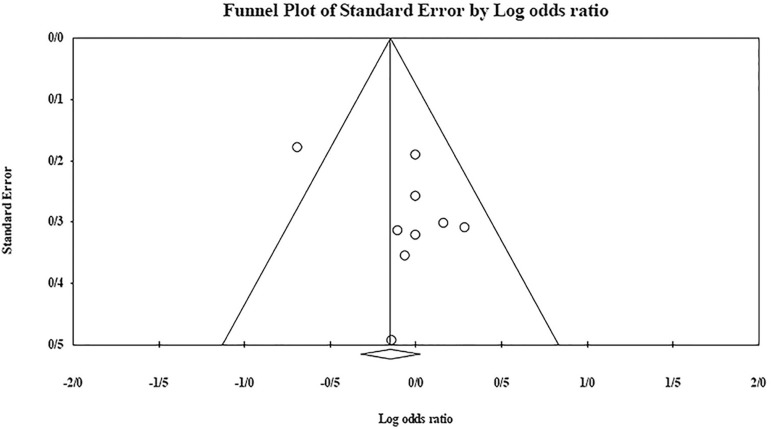
Funnel plot of KIR2DS4 shows a significant bias for Faridi et al. (2009).

**Figure 19:**
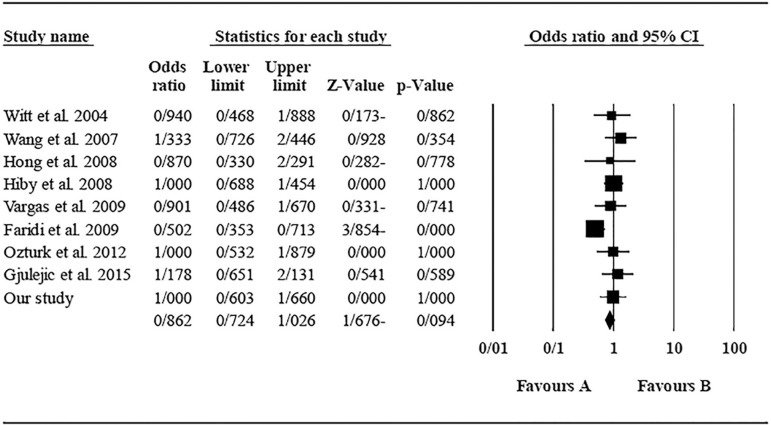
KIR2DS4 Forest plot (fixed).

**Figure 20:**
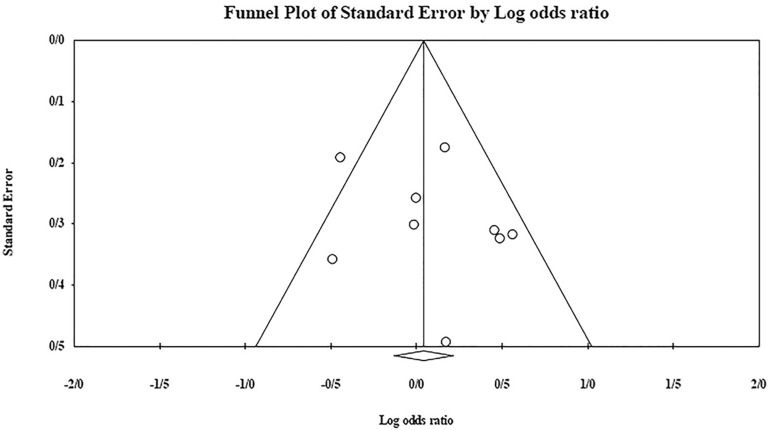
KIR2DS5 Funnel plot showing a significant bias for Hiby et al. (2008).

**Figure 21:**
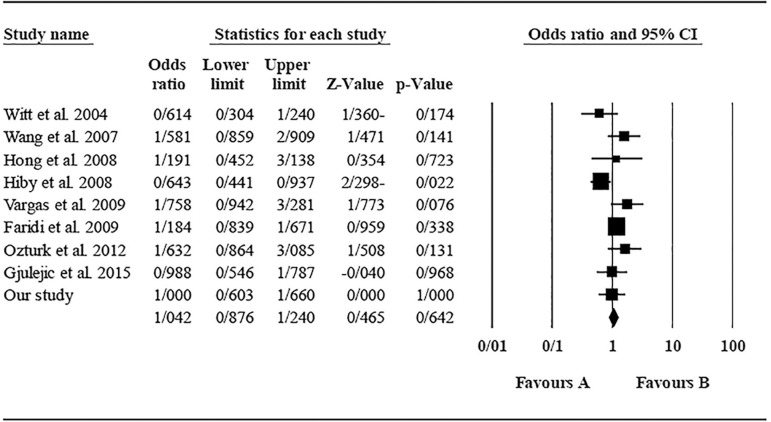
KIR2DS5 Forest plot (fixed).

**Figure 22:**
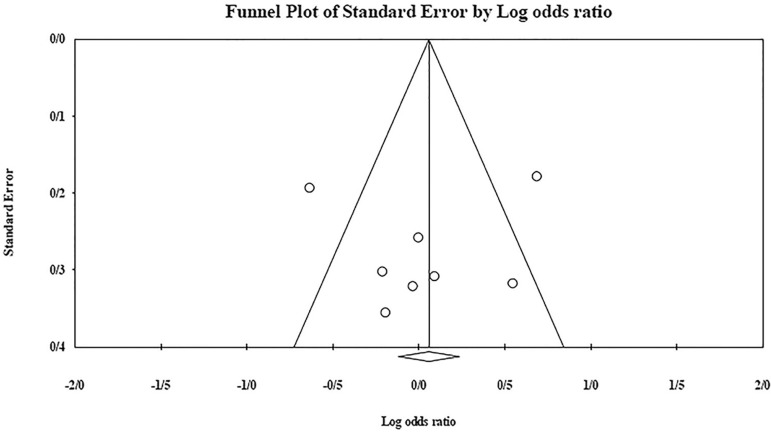
KIR3DS1 Funnel plot showing a significant bias for Hiby et al. (2008) and Faridi et al. (2009).

**Figure 23:**
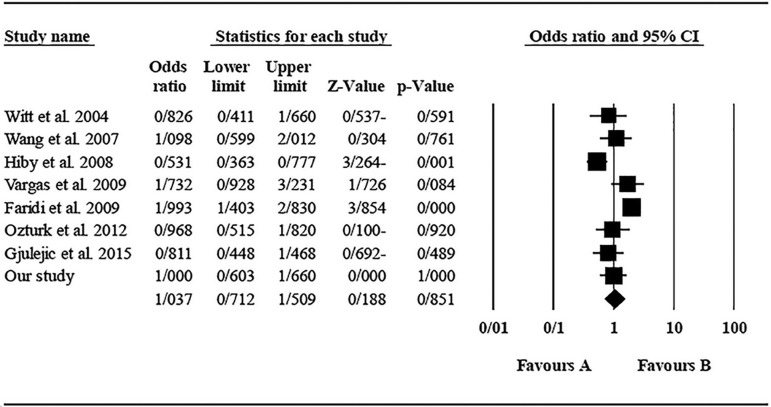
KIR3DS1 Forest plot (random).

The role of *KIR2DS4* in RSA was not statistically significant (*p*=0.094; OR=0.762; fixed). Faridi *et al.* (2009) showed significantly more protective effect of this gene in comparison to other studies (funnel plot *p* value <0.05) (Figures 18 and 19). The role of *KIR2DS5* in RSA was not statistically significant (*p*=0.642; OR=1.042; fixed). Hiby *et al*. (2008) showed a significantly more protective effect of this gene in comparison to other studies (funnel plot *p* value <0.05) (Figures 20 and 21). The role of *KIR3DS1* in RSA was not statistically significant (*p*=0.851; OR=1.037; random). Hiby *et al*. (2008) and Faridi *et al*. (2009) showed significantly more protective and risk effect of this gene in comparison to other studies, respectively (funnel plot *p* value <0.05) (Figures 22 and 23). 

## DISCUSSION

### Summary of evidence

NKs are lymphocytes that participate in the innate immune system. They have 2 subtypes: CD16^+^CD56^dim^ and CD16^-^CD56^bright^ that are called as cytotoxic and immune-regulatory NKs, respectively. In the implantation site, the NKs are mainly CD56^bright^. Hence, the immune system has a positive and protecting role in implantation and early pregnancy. Embryo implantation and pregnancy are a type of transplantation called semi-allograft. Thus, we need immune tolerance to have a successful pregnancy. The NKs play their roles with their KIRs interacting with the HLAs expressed on trophoblasts (Würfel, 2016). Because of the important roles of NKs in the implantation process, this meta-analysis aimed to investigate the role of maternal *KIR* genes diversity in RSA.

Among the investigated genes, only the results of *3DL1, 2DS2* and *2DS3* were statistically significant with protective, risk and risk effect impacts, respectively (Table 3). If we adjust multiple test correction for these findings, none of them would remain significant. It shows that there is no specific *KIR* gene predicting RSA. The funnel plot analyses showed that Faridi *et al*. (2009), in India, had more publication bias in comparison to the others. In our study we showed that maternal *KIR2DS1* in combination with paternal *HLA-C2* can be a risk factor (Akbari *et al*., 2018). 

**Table 3:** The pooled results of the meta-analsis. In the cases I2>50 random effect model has also been performed.

Pooled	Fixed effect			Random effect		
Gene	I2	P value	Odds ratio	I2	*p* value	Odds ratio
**2DL1**	20.92	0.051	0.849	-	-	-
**2DL2**	36.59	0.325	1.091	-	-	-
**2DL3**	0.00	0.448	1.069	-	-	-
**2DL4**						
**2DL5**	53.79	0.521	0.945	0.00	0.767	0.960
**3DL1**	47.16	0.044*	0.833	-	-	-
**3DL2**						
**3DL3**						
**2DS1**	70.31	0.990	0.999	0.00	0.726	1.058
**2DS2**	25.97	0.034*	1.195	-	-	-
**2DS3**	0.00	0.013*	1.246	-	-	-
**2DS4**	40.36	0.094	0.862	-	-	-
**2DS5**	48.52	0.642	1.042	-	-	-
**3DS1**	75.82	0.525	1.059	0.00	0.851	1.037
**2DP1**						
**3DP1**						

* significant at 0.05

### Literature review

This concern in reproductive immunology dates back to 2004. Witt *et al*. (2004) found no significant association of maternal *KIR* genes with the risk of RSA in a Brazilian population. Lack of paternal or fetal evaluation of *HLA-C* was their study limitation. Yamada *et al*. (2004) evaluated different immune markers such as CD94, CD158 (the very KIR) and CD161 through flow cytometry in 20 RSA women and 15 fertile controls. They found a lower level of CD158a (the very KIR2DL1) in the RSA group. Their low sample size was a limitation in their study (Yamada et al., 2004). Because of their quantitative approach and different aims and protocols, we excluded that study from our meta-analysis. Varla-Leftherioti *et al*. (2005) evaluated only *KIR2DL1, 2DL2* and *2DL3* among the *KIR* genes in a small sample size. Wang *et al*. (2007) found a risk association for *KIR2DS1* in a Chinese population. They evaluated *HLA-C* in couples, similar to our original experience. Conversely, our original study and some studies before, e.g. Hiby *et al*. (2008), found a strongly protecting association for *KIR2DS1* in a Caucasian population. However, since their control group criteria was to be a first-birth woman, this might be the reason of their publication bias. Vargas *et al.* (2009) found a risk association for the number of maternal activating *KIR* genes. Faridi *et al*. (2009) found that RSA was more associated with activating, and more protected with inhibitory *KIR* genes. Nowak *et al*. (2009) found that RSA could be associated with *KIR* genotypes. Conversely, other studies found that RSA was more frequent in patients with genotypes bearing 6 inhibitory genes. Because we did not have access to the frequencies of *KIR* genes, we excluded this study from our meta-analysis. Nowak *et al*. (2011) found that female heterozygosity for *HLA-C* in combination with *AA* KIR genotype could be a protecting factor for RSA. Khosravifar *et al*. (2011) investigated the role of maternal *KIR* and parental *HLA-C* in an Iranian population. They found that RSA was associated with maternal *HLA-C2*. Ozturk *et al*. (2012) found a protecting role for the KIR *AA* genotype. A small sample size and one miscarriage episode in the RSA group were the negative points of their study. Alecsandru *et al*. (2014) found that maternal *AA* genotype was a risk factor affecting the success of double embryo transformation. Djulejic *et al*. (2015) evaluated the role of *KIR* genes on women with any fertility problem. Hence, we excluded it from our meta-analysis. Nowak *et al*. (2016) investigated the role of *KIR2DL4* and *HLA-G* polymorphisms in RSA. Dambaeva *et al*. (2016) showed that maternal *KIR2DS1* is not a risk factor for RSA by itself, rather its combination with maternal *HLA-C2* could be associated. 

### Interpretation

As we observe above, there are many paradoxical findings for the role of maternal *KIR* genes in RSA. This can be justified through reasons like different ethnicities, different sample sizes, different RSA group criteria, different control criteria, and so on. In all the studies in Table 1, the genotyping method used was polymerase chain reaction with sequencing specific primers (PCR-SSP), and PCR with sequence specific oligonucleotides (PCR-SSO). Therefore, the genotyping method cannot be a reason for such paradoxes. Other features likely to be involved with this paradox are shown as a cluster analysis (Tables 1 and 2, Figure 1). 

The results of *KIR2DS1* had more publication bias based on funnel plots than the present meta-analysis. A paradoxical piece of evidence is that in early pregnancy KIR2DS1 is a helping factor (contrary to some studies), because its activating role (especially in combination with trophoblast HLA-C2) results in higher cytokine releasing of UNKs (Xiong *et al*., 2013). Hence, it seems that this receptor has a protecting role for implantation and placentation, and is a risk factor for late pregnancy maintenance. For instance, Alecsandru *et al*. (2014) found that maternal *AA* genotype was a risk factor for the success of assisted reproduction. *AA* is the most inhibitory genotype and therefore it supports this hypothesis that NK activation is necessary in early pregnancy. Pregnancy loss has numerous causes, in particular embryo genetic and chromosomal abnormalities. Therefore, the immune system's theoretical role is to reject such malformed embryos. Therefore, this risky role of activating KIRs is in fact a protecting role! Of course, it is remarkable that the lack of genetic evaluation of the lost embryos was a limitation for the studies imported to this meta-analysis. It is suggested that this variable should be adjusted in future studies. 

### Limitations

Although we found significant associations involving 3 genes in the meta-analysis (Table 3), but these findings would not be reliable, because, 1) the odds ratios are not large enough to show a remarkable effect size; 2) the paper selection and homogenizing process of meta-analyses are different and customized among researchers; 3) there were a lot of missed data even in the cited studies; 4) pregnancy loss has a number of definitions such as abortion, stillbirth (Gold *et al*., 2010) and assisted reproduction failure (Mitra & Boroujeni, 2015), and happens because due to conditions such as the anti-phospholipid syndrome (APS) (Rand *et al*., 1997), and there might be confusion involving these concepts. Adjusting models in future studies help researchers solve these limitations.

## CONCLUSION

The role of maternal *KIR* gene diversity in RSA is still in unclear, although our meta-analysis showed 3 genes as associated factors. *KIR3DL1* was a protecting factor, and *KIR2DS2* and *KIR2DS3*, which proved to be risk factors for RSA. For *KIR2DS1* there was a high heterogeneity. It seems that its role is different among different causes of pregnancy loss. Our previous case-control original investigation showed a significant relation with maternal *KIR2DS1* in combination with paternal *HLA-C2* as a risk factor. In order to clarify this role we have some suggestions for future studies, such as investigations of this combination concerning the success rate of assisted reproduction, for early first trimester abortions occurring after implantation and early placentation, for stillbirth groups, for abortions secondary to APS, and for successful and unsuccessful pregnancies of malformed embryos and fetuses. We would also like to suggest adjusting models and cohort studies. 
